# Sestrin2 is induced by glucose starvation via the unfolded protein response and protects cells from non-canonical necroptotic cell death

**DOI:** 10.1038/srep22538

**Published:** 2016-03-02

**Authors:** Boxiao Ding, Anita Parmigiani, Ajit S. Divakaruni, Kellie Archer, Anne N. Murphy, Andrei V. Budanov

**Affiliations:** 1 Department of Human and Molecular Genetics, Goodwin Research Laboratories, Massey Cancer Center, Virginia Commonwealth University, Richmond, VA 23298, USA; 2 Department of Biostatistics, Goodwin Research Laboratories, Massey Cancer Center, Virginia Commonwealth University, Richmond, VA 23298, USA; 3 Department of Pharmacology, University of California, San Diego, La Jolla, CA 92093, USA

## Abstract

Sestrin2 is a member of a family of stress responsive proteins, which controls cell viability via antioxidant activity and regulation of the mammalian target of rapamycin protein kinase (mTOR). Sestrin2 is induced by different stress insults, which diminish ATP production and induce energetic stress in the cells. Glucose is a critical substrate for ATP production utilized via glycolysis and mitochondrial respiration as well as for glycosylation of newly synthesized proteins in the endoplasmic reticulum (ER) and Golgi. Thus, glucose starvation causes both energy deficiency and activation of ER stress followed by the unfolding protein response (UPR). Here, we show that UPR induces Sestrin2 via ATF4 and NRF2 transcription factors and demonstrate that Sestrin2 protects cells from glucose starvation-induced cell death. Sestrin2 inactivation sensitizes cells to necroptotic cell death that is associated with a decline in ATP levels and can be suppressed by Necrostatin 7. We propose that Sestrin2 protects cells from glucose starvation-induced cell death via regulation of mitochondrial homeostasis.

Eukaryotic organisms rely on glucose as a critical source for ATP production when metabolized via glycolysis and mitochondrial respiration. Glucose is also a substrate for glycosylation, a post-translational modification that occurs primarily in the endoplasmic reticulum (ER)^1^. Glucose starvation activates at least two mechanisms of the stress response: one senses energy availability via activation of 5′-AMP-activated protein kinase (AMPK)^2^, and another is activated through accumulation of unfolded and unprocessed proteins in the ER and induction of ER stress followed by a program called the unfolded protein response (UPR)^3^,^4^. The UPR activates three pathways mediated by: protein kinase (PKR)-like ER kinase (PERK1), activating transcription factor 6 (ATF6) and inositol-requiring enzyme 1 (IRE1)^3^,^5^. PERK1 directly phosphorylates and inhibits eukaryotic translation initiation factor 2 alpha (eIF2α), causing suppression of global protein synthesis; however, it also re-directs the translational machinery toward translation of specific mRNAs involved in the UPR^4^,^5^.

The major function of the PERK1-eIF2α pathway is to activate transcription factor 4 (ATF4)^3^, which is induced via a translation-dependent mechanism. ATF4 is a master regulator of numerous genes involved in the UPR^6^. Some of these genes, such as transcription factor CHOP, induce cell death, while others protect cell viability through suppression of cell death machinery and relief of ER stress, or by regulating metabolism^4^. Another important target of PERK is the master regulator of antioxidant response and metabolism Nuclear factor (erythroid-derived 2)-like 2 (NRF2)^7^. Under non-stressed conditions NRF2 is constantly bound to its partner Kelch like-ECH-associated protein 1 (Keap1) which retains NRF2 in the cytoplasm and stimulates its degradation. Under stress conditions, PERK directly phosphorylates NRF2 leading to its dissociation from Keap1 and translocation to the nucleus where it activates the transcription of its target genes via recognition of antioxidant responsive elements (ARE)^8^.

We have identified and characterized the Sestrin (SESN) family of stress-responsive genes^9^,^10^ composed of *SESN1, SESN2* and *SESN3* genes in mammals while only one Sestrin ortholog has been found in invertebrates^10^. Sestrins are activated by multiple insults including oxidative stress, DNA damage, hypoxia, growth factor depletion and ER stress^11^. We demonstrated that protein products of Sestrin genes work as antioxidant proteins suppressing oxidative DNA damage and mutagenesis^12^,^13^. Furthermore, Sestrins also inhibit mammalian target of rapamycin (mTOR) complex 1 (mTORC1) kinase, a critical regulator of cell growth and metabolism^14^,^15^,^16^. Sestrins inhibit mTORC1 in a manner dependent on AMPK and tuberous sclerosis complex (TSC), which, in turn, inhibits the small GTPase Rheb, a critical activator of mTORC1^14^,^15^,^17^,^18^,^19^. We and others have also described a parallel mechanism of mTORC1 inhibition by Sestrins mediated by small Rag GTPases^20^,^21^,^22^. Active forms of RagA/B:RagC/D heterodimers bring mTORC1 to the lysosomes where it interacts with Rheb^23^. The RagA/B activity is inhibited by its GTPase activated protein (GAP) - GATOR1 protein complex, which is in turn inhibited by GATOR2 protein complex. Sestrins interact with GATOR2 and inhibit mTORC1 lysosomal localization^20^,^21^.

In our previous publications, we demonstrated that SESN2 is activated in response to some metabolic stress factors and is involved in the regulation of cell viability^9^,^24^; however, the precise role of SESN2 in the regulation of cell death is not well established. Here we show that glucose starvation stimulates SESN2 via induction of ER stress and that SESN2 protects cells from necrotic cell death through the support of cell metabolism, ATP production and mitochondrial function.

## Results

### SESN2 is activated in response to energy stress in a manner similar to the UPR induction

**Figure 1:**
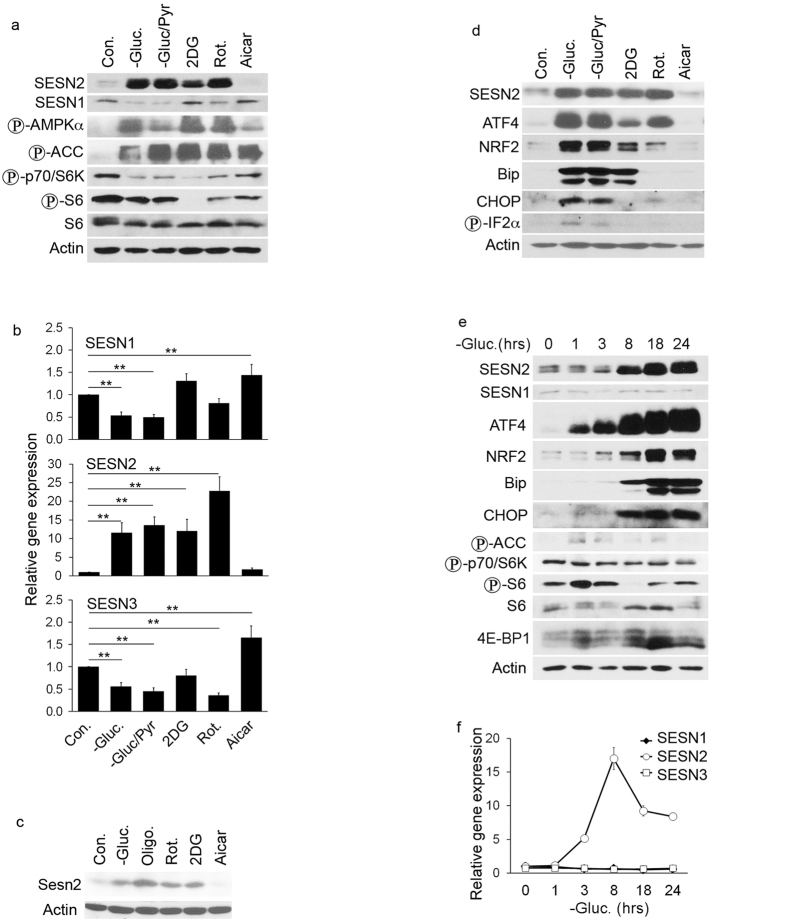
SESN2 is activated by glucose starvation in manner correlated with the induction of the unfolded protein response. (**a,b**) Energetic stress, but not AICAR treatment stimulates SESN2 expression. H1299 cells were treated with glucose-free medium in the presence or absence of pyruvate, rotenone (20 μM), 2-deoxyglucose (2DG) (2.5 mM) or Aicar (1 mM) for 12 hr. The phosphorylation of the components of the AMPK-mTORC1 pathway and the expression of Sestrin family members were analyzed by immunoblotting with the indicated antibodies (**a**) or quantitative real time PCR (qPCR) (**b**). The data represent a mean of three independent experiments ± S.D. (**b**) statistical analysis: one-way ANOVA followed by comparisons to the control group with Bonferroni correction (adjusted α = 0.05/5 = 0.01, **P < 0.01). (**c**) Energetic stress, but not Aicar treatment, stimulates Sesn2 expression in MEF. MEF were treated and analyzed as in (**a**). (**d–f**) Activation of SESN2 correlates with activation of the UPR proteins. H1299 cells were incubated with glucose-free medium for different time intervals and expression of SESN2 and the UPR proteins and phosphorylation of components of the AMPK-mTORC1 pathway were analyzed by immunoblotting (**d,e**) and qPCR (**f**).

Different inducers of energy stress such as an inhibitor of glucose metabolism - 2-deoxyglucose (2DG), an inhibitor of complex I of the mitochondrial electron transport chain - rotenone and hypoxia stimulate expression of SESN2^9^,^20^,^24^. Thus, we theorized that any type of stress associated with diminished ATP may stimulate SESN2 expression, and that AMP itself may trigger SESN2 induction. To test this possibility, we treated cells with 2DG, rotenone, glucose-free medium with and without sodium pyruvate, or Aicar (an AMP analog), and compared the effects of each of these treatments on the activation of Sestrins as measured by immunoblotting and quantitative real time PCR (qPCR) in H1299 cells and in immortalized mouse embryonic fibroblasts (MEF). Glucose withdrawal, 2DG and rotenone activated SESN2; however, Aicar treatment had no effect on SESN2 induction (Fig. 1a–c). Analysis of AMPK activation by examination of phosphorylation of AMPK and its target ACC showed that all treatments including Aicar strongly activated AMPK and inhibited mTORC1-dependent phosphorylation of S6, indicating that regulation of AMPK-mTORC1 pathway is probably not the trigger of SESN2 activation under conditions of energy deficiency. We also observed that SESN2 was the only Sestrin family member to be activated in our experimental conditions, indicating that SESN2 is the major responder to energy stress among Sestrins (Fig. 1a,b).

Proper protein folding in the ER requires ATP. Therefore, low ATP levels might trigger ER stress and UPR through accumulation of misfolded proteins. We observed that all treatments except Aicar promoted accumulation of ER stress inducible transcription factors ATF4 and NRF2 (Fig. 1d). The magnitude of UPR induction varied in different treatment conditions, and we observed the strongest activation of the hallmarks of ER stress such as Bip, CHOP and phosphorylation of eIF2α in the glucose-starved cells (Fig. 1d). Therefore, in subsequent studies we focused on the regulation of SESN2 by glucose starvation as the type of energetic stress. To confirm that activation of SESN2 correlates with induction of ER stress, we analyzed expression levels of SESN2 at different time points after glucose withdrawal and observed a clear correlation with the induction of SESN2, ATF4 and NRF2 as determined by immunoblotting and qPCR (Fig. 1e,f).

### Glucose starvation activates SESN2 via a mechanism dependent on ATF4 and NRF2, but not p53

**Figure 2:**
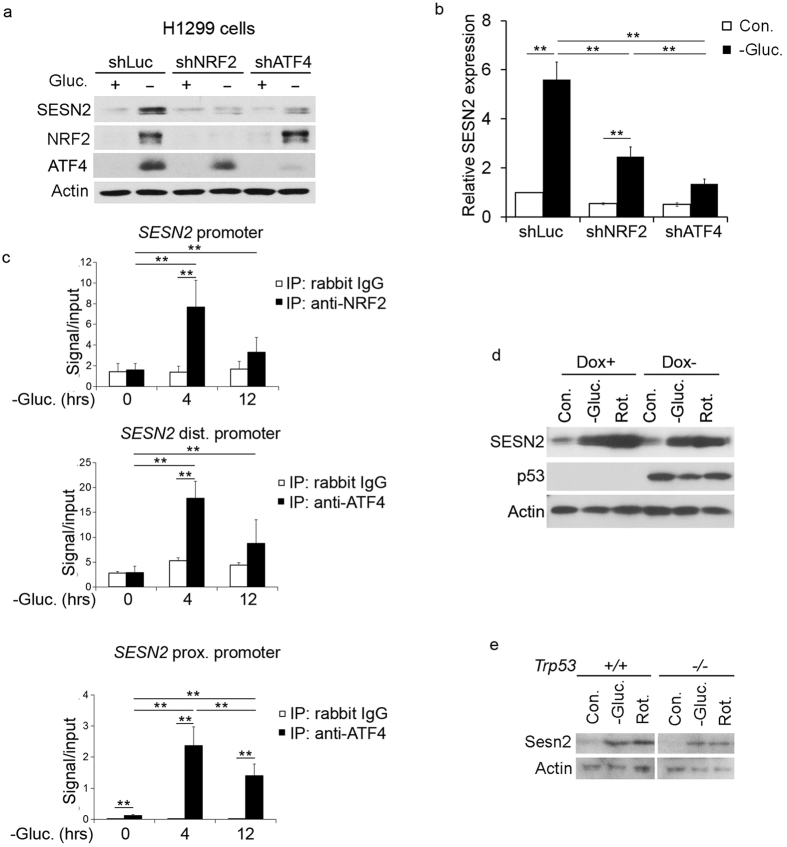
Glucose starvation activates SESN2 via NRF2- and ATF4- dependent but via p53-independent mechanism. (**a,b**) Silencing of either NRF2 or ATF4 inhibits SESN2 activation by glucose withdrawal. H1299 cells were infected with lentiviral vectors expressing shNRF2, shATF4 or control shLuciferase (shLuc). The cells were incubated with glucose-free medium for 12 hr and expression of the corresponding proteins was examined by immunoblotting (**a**) and qPCR (**b**). The data represent a mean of three independent experiments ± S.D. Data in (**b**) were analyzed with two-way ANOVA followed by linear contrasts with Bonferroni correction (adjusted α = 0.05/9 = 0.0056, **P < 0.0056). (**c**) NRF2 and ATF4 bind corresponding NRF2- and ATF4-responsive elements in the *SESN2* promoter. Cells were incubated in glucose-free medium for 4 and 12 hours and NRF2 and ATF4 binding to the corresponding promoter elements were analyzed by CHIP assay (dist.-distal and prox.-proximal ATF4-responsive elements in *SESN2* promoter). The data represent a mean of three independent experiments ± S.D. (**c**) statistical analysis: two-way ANOVA followed by linear contrasts with Bonferroni correction (adjusted α = 0.05/6 = 0.0083, **P < 0.0083). (**d,e**) p53 does not play a significant role in SESN2 regulation by energetic stress. (**c**) H1299 cells with doxycycline-inducible expression of p53 were treated with glucose-free medium or rotenone (20 μM) and the expression of the indicated proteins was analyzed by immunoblotting. (**d**) *Trp53*^+/+^ and *Trp53*^−/−^ mouse embryonic fibroblasts were incubated in glucose-free medium or with rotenone (20 μM) for 12 hr and the expression of the corresponding proteins was analyzed by immunoblotting.

To study the role of ATF4 and NRF2 in the activation of SESN2 in response to glucose starvation, we silenced each of these proteins by shRNA lentivirus, treated cells with glucose-free medium and analyzed protein and mRNA expression. Silencing either NRF2 or ATF4 prevented the activation of SESN2 upon glucose starvation (Fig. 2a,b). To study whether glucose starvation can stimulate binding of NRF2 to NRF2-binding element in position −550^25^ and ATF4 to its cognate responsive elements in proximal (−138) and distant (−16 kB) parts of the *SESN2* promoter^26^, we performed chromatin immunoprecipitation assay (CHIP). We observed a strong interaction of NRF2 and ATF4 with the *SESN2* promoter in response to glucose starvation (Fig. 2c). Another transcription factor that plays a major role in the regulation of SESN2 is p53, and energy stress is known to trigger p53 activation^9^,^27^. As H1299 cells do not express p53, we utilized H1299-tta cells with doxycycline-dependent p53 regulation (H1299-tet-off-p53) in order to analyze the potential contribution of p53 in SESN2 induction by glucose withdrawal^9^. As shown in Fig. 2d, glucose starvation, as well as rotenone treatment, induced SESN2 in a similar manner in the p53-positive and p53-negative cells. In a complementary experiment we treated *Trp53*^+/+^ and *Trp3*^−/−^ MEF with glucose-free medium and rotenone and observed a negligible effect of p53 on mouse Sesn2 activation (Fig. 2e). Taken together, these data indicate that the transcription factors ATF4 and NRF2, but not p53, are responsible for SESN2 induction in glucose-starved cells.

### SESN2 is not a critical regulator of ER stress and AMPK activation in response to glucose starvation

**Figure 3:**
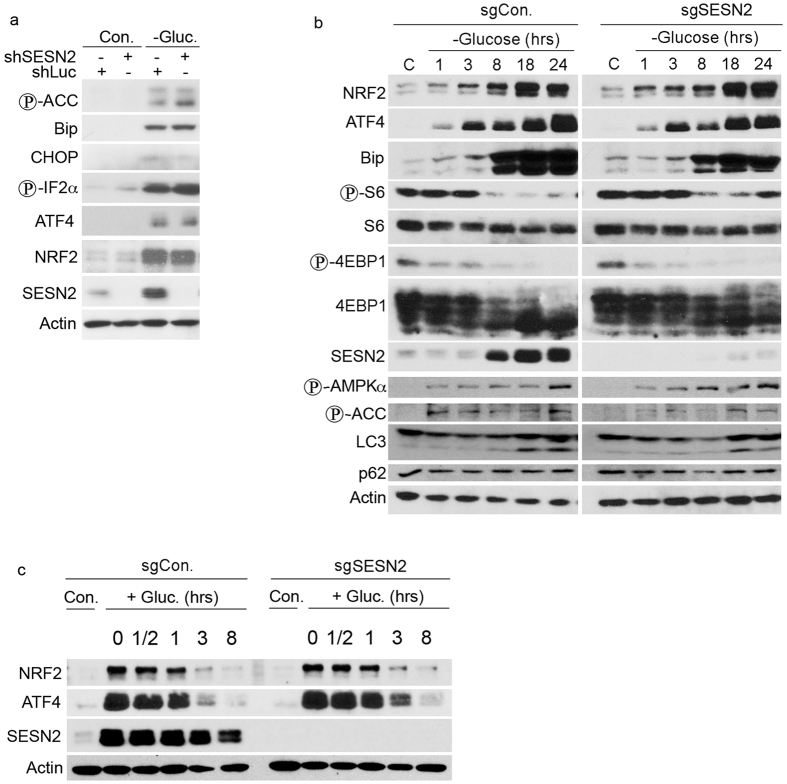
SESN2 does not play a role in regulation of the AMPK-mTORC1 pathway and UPR during glucose starvation. (**a**) Silencing of SESN2 by shSESN2 lentivirus does not affect the magnitude of ACC phosphorylation and UPR activation. SESN2-silenced and control shLuc-expressed H1299 cells were incubated with glucose-free medium and ACC phosphorylation and activation of the UPR proteins were assessed by immunoblotting. (**b**) SESN2 does not affect the kinetics of UPR activation and regulation of the AMPK-mTORC1 pathway by glucose starvation. sgSESN2- or sgCon.- H1299 cells were incubated with glucose-free medium for different time intervals and the phosphorylation and expression of the corresponding proteins were analyzed by immunoblotting. (**c**) SESN2 does not affect UPR release following re-feeding of cells with glucose. Cells as in (**b**) were incubated for 12 hr with glucose-free medium and re-supplied with glucose for different time intervals. The expression of corresponding proteins was analyzed by immunoblotting.

As previously reported, SESN2 is a potential regulator of the AMPK-mTORC1 pathway and ER stress response^14^,^28^,^29^. To study whether SESN2 plays a role in regulation of ACC phosphorylation or expression of ER stress-induced proteins, indicators of the severity of ER stress, we silenced SESN2 in H1299 cells by shRNA lentivirus and analyzed ACC phosphorylation and activation of ER stress proteins by immunoblotting. We observed similar levels of ACC phosphorylation and activation of Bip, CHOP, IF2α, ATF4, and NRF2 in glucose-starved control and SESN2-silenced cells (Fig. 3a). We also analyzed the kinetics of activation of UPR factors and phosphorylation of AMPK, ACC and mTORC1 targets S6 and 4EBP1 in the control cells and in the cells, where SESN2 was inactivated by a CRISPR construct. No significant difference was found in the activation of NRF2, ATF4, Bip, AMPK, and ACC or in the inhibition of S6 and 4EBP1 phosphorylation (Fig. 3b). Additionally, we measured accumulation of the pro-autophagic LC3-II protein and the expression of autophagic p62 protein and did not observe any noticeable difference. This indicates that SESN2 does not play an important role in regulation of the UPR or AMPK-mTORC1 under glucose starvation (Fig. 3b). We also considered the possibility that SESN2 can provide relief from ER stress when cells are re-supplied with glucose. Thus, we analyzed NRF2 and ATF4 protein levels at different time points after the cells were re-fed with glucose and observed that both proteins were downregulated with similar kinetics in both SESN2-proficient and SESN2-deficient cells (Fig. 3c).

### Sesn2 protects from cell death induced by glucose starvation

**Figure 4:**
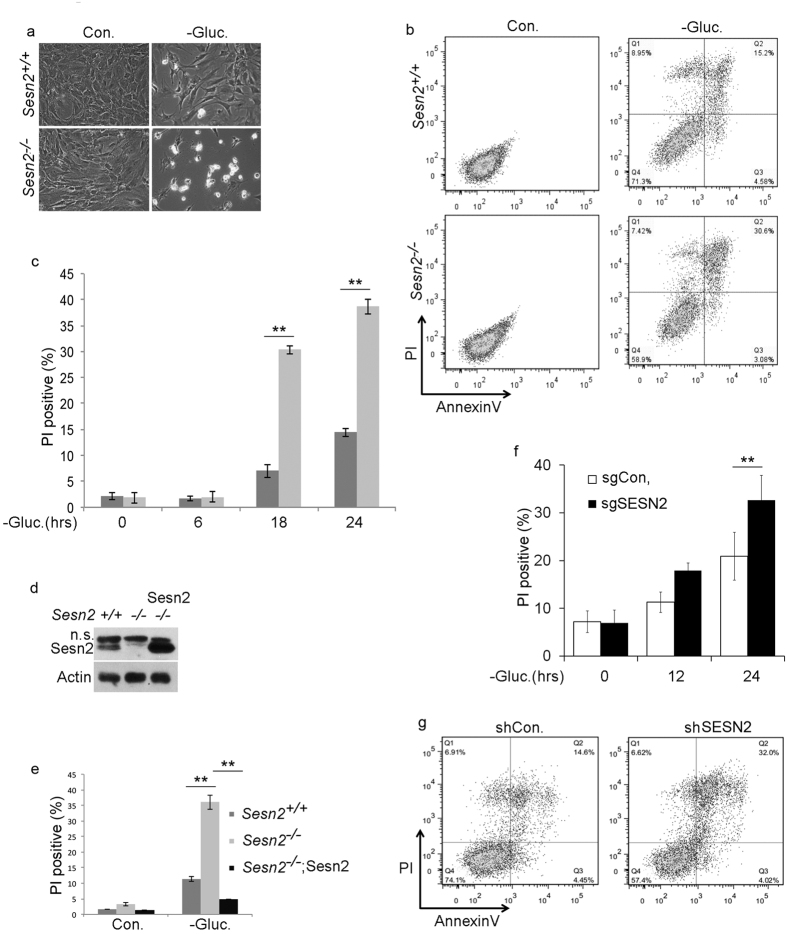
Sesn2 protects cells against cell death induced by glucose withdrawal. (**a–c**) *Sesn2*^−/−^ cells are more susceptible to cell death in response to glucose starvation than their *Sesn2*^+/+^ counterparts. (**a**) Phase-contrast microscopy of *Sesn2*^+/+^and *Sesn2*^−/−^ MEF incubated with control and glucose-free medium for 24 hr. (**b**) *Sesn2*^+/+^ and *Sesn2*^−/−^ MEF were incubated with glucose-free medium for 24 hr and cell death was determined by Annexin V/PI staining followed by flow cytometry analysis. (**c**) *Sesn2*^+/+^ and *Sesn2*^−/−^ MEF were incubated with glucose-free medium for different time intervals and cell death was determined by PI staining followed by flow cytometry analysis. The data represent a mean of three independent experiments ± S.D. Two-way ANOVA followed by linear contrasts with Bonferroni correction (adjusted α = 0.05/4 = 0.0125, **P < 0.0125). (**d,e**) Re-constitution of Sesn2 in Sesn2-deficient cells suppresses cell death induced by glucose starvation. (**d**) *Sesn2*^−/−^ MEF were infected with retroviral pBabe-hygro construct expressing Sesn2 and selected with hygromycin. Sesn2 expression was analyzed by immunoblotting. (**e**) Cells from (**d**) were incubated with glucose-free medium and analyzed by PI staining as in (**b**). The data represent a mean of three independent experiments ± S.D. Two-way ANOVA followed by linear contrasts with Bonferroni correction (adjusted α = 0.05/6 = 0.0083, **P < 0.0083). (**f**) sgCon. and sgSESN2 H1299 cells were treated with glucose-free medium and analyzed as in (**b**). The data represent a mean of three independent experiments ± S.D. Two-way ANOVA followed by linear contrasts with Bonferroni correction (adjusted α = 0.05/3 = 0.0167, **P < 0.0167). (**g**) H460 cells expressed shSESN2 or control shLuc constructs were incubated with glucose-free medium for 24 hr and analyzed as in (**b**).

Glucose is required for cellular homeostasis, and prolonged glucose starvation induces cell death^30^. Since SESN2 plays an important role in the regulation of cell viability under stress^9^, we investigated its role in regulating cell viability during glucose withdrawal. We treated immortalized *Sesn2*^+/+^ and *Sesn2*^−/−^ MEF with glucose-free medium and analyzed cell death by AnnexinV/-PI staining. Following glucose deprivation, the loss in viability was more than doubled at 24 hr in Sesn2-deficient MEF compared to their WT counterparts (Fig. 4a–c). Interestingly, most of cells were AnnexinV^+^PI^+^ or PI^+^, while we observed only a negligible fraction of AnnexinV^+^PI^−^ cells between *Sesn2*^+/+^ and *Sesn2*^−/−^ cell types, even at early time points after glucose withdrawal. This suggests that most of the cells died via necrosis/necroptosis or quickly progressed from apoptosis to necrosis (Fig. 4b). To validate the role of Sesn2 in protection against glucose starvation-induced cell death we re-constituted *Sesn2*^−/−^ MEF with a retrovirus construct expressing Sesn2 (Fig. 4d) and analyzed cell death after glucose withdrawal. We observed that Sesn2 re-constitution strongly suppressed cell death in the Sesn2-deficient MEF (Fig. 4d,e). To study whether Sesn2 has some protective effects in cancer cells, we incubated lung adenocarcinoma SESN2-deficient H1299 cells or SESN2-silenced H460 cells and their control counterparts with glucose-free medium and found that in both cell lines SESN2 had a significant protective effect against cell death (Fig. 4f,g).

### Sesn2 protects against glucose starvation-induced cell death not through regulation of caspase-dependent apoptosis, ROS or mTORC1

**Figure 5:**
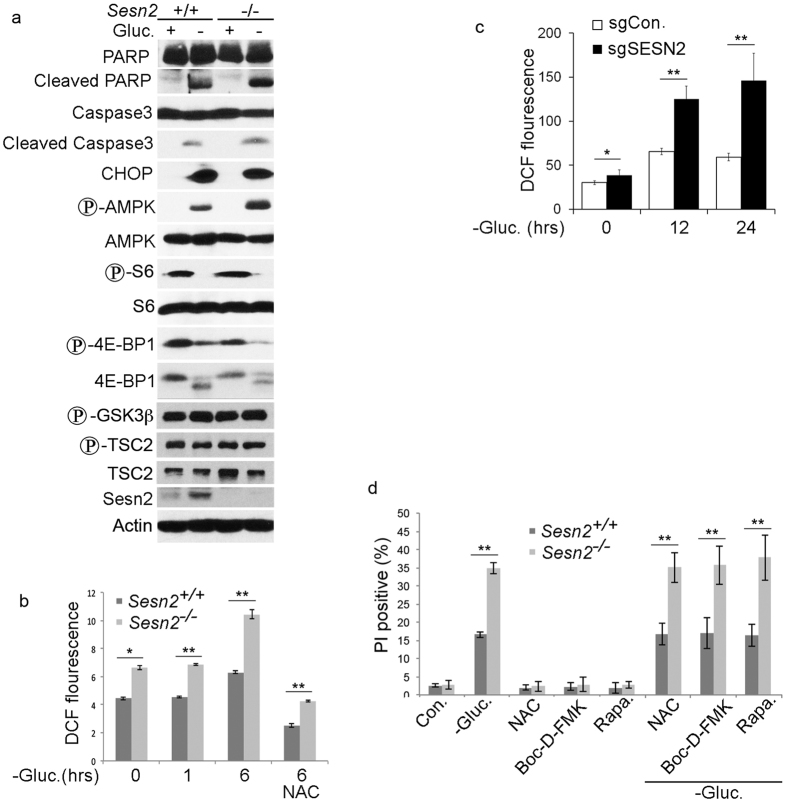
Sesn2 suppresses cell death induced by glucose starvation via a mechanism independent on caspase activation, ROS or mTORC1. (**a**) Sesn2 deficiency does not affect caspase activation and regulation of the AMPK-mTORC1 pathway by glucose starvation. *Sesn2*^+/+^ and *Sesn2*^−/−^ MEF were incubated with control high-glucose (gluc+) or glucose-free (gluc−) medium for 24 hr and the expression and phosphorylation of the corresponding proteins were analyzed by immunoblotting with the indicated antibodies. (**b,c**) Sesn2-deficient cells have higher levels of ROS in normal and glucose-free medium. (**b**) *Sesn2*^+/+^ and *Sesn2*^−/−^ MEF were incubated with glucose-free medium for different time intervals and the levels of ROS were determined by DCFDA staining followed by flow cytometry. The data represent a mean of three independent experiments ± S.D. Two-way ANOVA followed by linear contrasts with Bonferroni correction (adjusted α = 0.05/5 = 0.01, **P < 0.01). (**c**) sgCon. and sgSESN2 H1299 cells were incubated with glucose-free medium and analyzed as in (**b**). Two-way ANOVA with replication, followed by Bonferroni post-test (*p < 0.05, **p < 0.01). (**d**) Treatment with antioxidant, pan-caspase inhibitor or mTORC1 inhibitor does not affect cell death induced by glucose starvation. *Sesn2*^+/+^ and *Sesn2*^−/−^ MEF were treated with antioxidant N-acethyl-cysteine, NAC (5 mM), inhibitor of caspases, Boc-D-FMK (20 μM), or mTORC1 inhibitor, rapamycin (10 nM), for 24 hr and cell death levels were determined by PI staining followed by flow cytometry. The data represent a mean of three independent experiments ± S.D. Two-way ANOVA followed by linear contrasts with Bonferroni correction (adjusted α = 0.05/10 = 0.005, **P < 0.005).

As it was reported that Sesn2 protects from apoptosis under some stress conditions^24^,^28^, we analyzed the cleavage of caspase 3 and PARP, the markers of apoptotic cell death, after 24 hr of incubation with glucose-free medium. We observed no significant difference in cleavage of both proteins in *Sesn2*^+/+^ and *Sesn2*^−/−^ MEF indicating that the mode of cell death and protection by Sesn2 in response to glucose withdrawal is not through apoptosis (Fig. 5a). We also did not see any difference in regulation of CHOP, the major activator of apoptosis in response to ER stress^31^. As demonstrated previously Sesn2 can protect against cell death via regulation of the AMPK-mTORC1 pathway or suppression of reactive oxygen species^32^. To study whether Sesn2 regulates AMPK and mTORC1 in MEF in response to glucose starvation, we analyzed phosphorylation of AMPK, S6 and 4EBP1 and found that glucose starvation stimulates AMPK phosphorylation and inhibits S6 and 4EBP1 phosphorylation in a Sesn2-independent manner (Fig. 5a). It was also reported previously that Sesn2 might stimulate AKT protein kinase that inhibits cell death. To address this possibility, we analyzed AKT-dependent phosphorylation of GSK3α and TSC2 and did not find any differences in phosphorylation of these proteins between *Sesn2*^+/+^ and *Sesn2*^−/−^ MEF (Fig. 5a). We also analyzed the intracellular levels of ROS by DCF-DA staining in *Sesn2*^+/+^ and *Sesn2*^−/−^ MEF and in SESN2-proficient and SESN2-deficient H1299 cells incubated in glucose-free medium. As reported previously^12^,^13^, Sesn2-deficient cells showed higher levels of ROS as compared to the Sesn2-expressing controls at baseline. Glucose starvation led to accumulation of ROS in both Sesn2-proficient and Sesn2-deficient cells, although this phenomenon was more dramatic in the Sesn2-deficient cells (Fig. 5b,c). Treatment of glucose-starved cells with the ROS scavenger N-acetyl-cysteine (NAC) reduced ROS levels in both *Sesn2*^+/+^ and *Sesn2*^−/−^ MEF, but the difference in ROS levels between the *Sesn2*^+/+^ and *Sesn2*^−/−^ cells remained significant (Fig. 5b). To study whether Sesn2-controlled cell death in response to glucose starvation mediated by regulation of caspase activation, mTORC1 activity or ROS production, we incubated *Sesn2*^+/+^ and *Sesn2*^−/−^ MEF in glucose-free medium in the presence of the pan-caspase inhibitor, Boc-D-FMK, the mTORC1 inhibitor, rapamycin, or ROS scavenger, NAC. Cell death analysis revealed no difference between cells treated with glucose-free medium in the presence or absence of any of these inhibitors (Fig. 5d). The difference between *Sesn2*^+/+^ and *Sesn2*^−/−^ cells was preserved in all treatments, indicating that Sesn2 does not suppress cell death induced by glucose starvation by regulating caspases, mTORC1 or ROS.

### Sesn2 selectively protects cells from glucose starvation-induced cell death via a non-canonical necroptotic pathway

**Figure 6:**
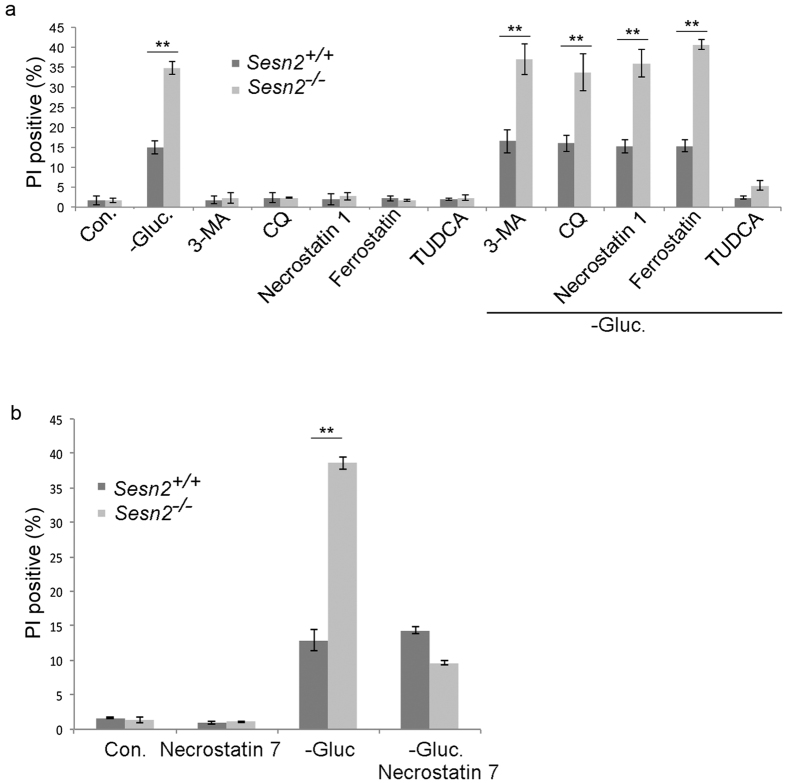
Glucose starvation-induced cell death is inhibited by Necrostatin 7. (**a**) Inhibitors of autophagy, necroptosis, or ferroptosis do not inhibit cell death induced by glucose starvation. *Sesn2*^+/+^ and *Sesn2*^−/−^ MEF were incubated in glucose free medium in the presence of inhibitors of autophagy, 3 MA (2 mM) or cloroquine, CQ (50 μM), an inhibitor of RIP1-dependent necroptosis, Necrostatin 1 (10 μg/ml), an inhibitor of ferroptosis, Ferrostatin (2 μM), or an inhibitor of ER stress, TUDCA (10μM). Cell death was analyzed by PI staining followed by flow cytometry. The data represent a mean of three independent experiments ± S.D. Two-way ANOVA followed by linear contrasts with Bonferroni correction (adjusted α = 0.05/16 = 0.003125, **P < 0.003125). (**b**) Cell death induced by glucose starvation is suppressed by Necrostatin 7 in *Sesn2*^−/−^ cells. *Sesn2*^+/+^ and *Sesn2*^−/−^ MEF were incubated with glucose-free medium in the presence or absence of Necrostatin 7 (5 μg/ml), and the levels of cell death were assessed by PI staining followed by flow cytometry. The data represent a mean of three independent experiments ± S.D. Two-way ANOVA with replication, followed by Bonferroni post-test (*p < 0.05, **p < 0.01).

Although apoptosis is the major form of physiological cell death, other types of cell death such as autophagic cell death, necroptosis and ferroptosis have been also reported^33^,^34^. To analyze whether glucose starvation induces cell death via any of these mechanisms and to determine the impact of Sesn2 on these processes, we incubated *Sesn2*^+/+^ and *Sesn2*^−/−^ MEF with glucose-free medium in the presence of autophagy inhibitors 3-Methyladenine (3-MA) or Chloroquine (CQ), an inhibitor of classical RIP1-dependent necroptosis - Necrostatin 1^35^, or an inhibitor of ferroptosis - Ferrostatin 1^36^. We observed no difference in cell death, whether cells were incubated in glucose-free medium in the presence or absence of any of these inhibitors, and the difference in cell death between *Sesn2*^+/+^ and *Sesn2*^−/−^ MEF was preserved in all of these experimental conditions (Fig. 6a). This indicates that in our experimental setting, Sesn2 does not protect cells from cell death either through regulation of autophagy, through classical RIP1-dependent necroptosis, or through ferroptosis. Interestingly, treatment with TUDCA, which works as an ER chaperone and mitigates some aspects of ER stress^37^, strongly inhibited cell death in both *Sesn2*^+/+^ and *Sesn2*^−/−^ cells (Fig. 6a), indicating that this type of cell death is relevant to the induction of ER stress.

In order to examine potential alternative forms of cell death inhibited by Sesn2, we treated cells with Necrostatin 7, which inhibits a form of necroptosis that proceeds via a RIP1-independent mechanism^38^. We observed that while Necrostatin 7 had no effect on cell death in control cells, it strongly inhibited cell death in *Sesn2*^−/−^ MEF, thus indicating that Sesn2 selectively protects from a non-canonical form of necroptosis (Fig. 6b).

### Sesn2 inhibits glucose starvation-induced cell death via preservation of cellular energy metabolism

**Figure 7:**
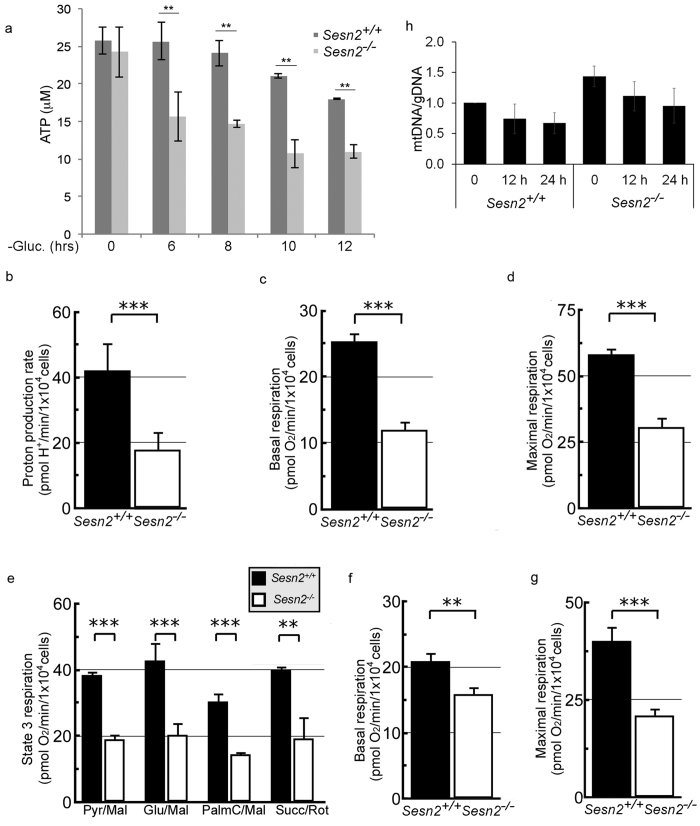
Sesn2 protects cells from cell death induced by glucose starvation via regulation of mitochondrial function. (**a**) Sesn2 supports ATP production in response to glucose starvation. *Sesn2*^+/+^ and *Sesn2*^−/−^ MEF were incubated with glucose-free medium and ATP levels were analyzed by an ATP determination kit. The lysates were normalized according to protein content and the values represent the concentration of ATP in the lysates. The data represent a mean of three independent experiments ± S.D. Two-way ANOVA followed by linear contrasts with Bonferroni correction (adjusted α = 0.05/5 = 0.01, **P < 0.01). (**b–g**) Mitochondrial activity is diminished in Sesn2-deficient MEF. *Sesn2*^−/−^ MEF have decreased proton production rate (**b**), rate of basal mitochondrial respiration (**c**) and maximal respiration rate in the presence of protonophore FCCP (**D**) as compared to *Sesn2*^+/+^ counterparts. (**e**) Sesn2-deficiency compromises the capacity of mitochondria to oxidize particular respiratory substrates including pyruvate, glutamate, or palmitoyl carnitine in the presence of malate (Pyr/Mal, Glu/Mal and PalmC/Mal), or succinate in the presence of rotenone (Succ/Rot) in the permeabilized cells. (**f,g**) *Sesn2*^−/−^ cells have a decreased rate of basal (**f**) and maximal mitochondrial respiration rate (**g**) as compared to *Sesn2*^+/+^ controls in glucose-free medium. (**b–g**) The results were normalized according to cell number and data presented as a mean of 6 biological replicates. The data are presented as mean ± S.D. At least 10 technical replicates (10 wells) were conducted for each biological replicate. Two-way t-test (**p < 0.05, ***p < 0.001). (h) *Sesn2*^−/−^ MEF have decreased mtDNA/gDNA ratio as compared to *Sesn2*^+/+^ cells. *Sesn2*^+/+^ and *Sesn2*^−/−^ cells were incubated with control and glucose free medium and the relative levels of mitochondrial (mtDNA) and genomic DNA (gDNA) were examined by qPCR. The data represent a mean of three independent experiments ± S.D. Two-way ANOVA followed by linear contrasts with Bonferroni correction (adjusted α = 0.05/7 = 0.007).

Necrosis/necroptosis is often associated with mitochondrial dysfunction and a drop in ATP levels. Because Sesn2 can regulate ATP production^24^, we studied whether Sesn2 can support ATP production in cells cultured in glucose-free medium. We analyzed ATP levels in *Sesn2*^+/+^ and *Sesn2*^−/−^ MEF and found that a drop in ATP levels was much faster in *Sesn2*^−/−^ cells as compared to *Sesn2*^+/+^ controls (Fig. 7a).

As Sesn2 clearly protects from ATP depletion associated with glucose deprivation, we used the Seahorse platform to assess the effect of Sesn2 expression on cellular energy metabolism and mitochondrial respiration. Assaying intact MEF, we discovered that the basal rate of extracellular acidification, expressed as the proton production rate (Fig. 7b), was significantly lower in Sesn2-deficient cells compared to control. The rate of extracellular acidification is primarily a function of glycolytic lactate production, along with contribution by TCA cycle generation of CO_2_ that produces carbonic acid^39^. Further, the basal (endogenous) rates of respiration were also significantly lower in the *Sesn2*^−/−^ cells (Fig. 7c). A significant difference in respiratory rate persisted after the addition of the respiratory uncoupler, carbonyl cyanide-*4*-(trifluoromethoxy)phenylhydrazone (FCCP) (Fig. 7d). FCCP stimulates the maximal rate of mitochondrial respiration indicating the respiratory capacity of mitochondria. These data imply a significant decrease in glycolytic and mitochondrial function in the *Sesn2*^−/−^ MEF. To study whether the effect of Sesn2 on mitochondrial function is on a particular oxidative pathway within mitochondria, we measured rates of State 3 (ADP-stimulated) respiration in permeabilized fibroblasts. Rates of respiration on pyruvate/malate, glutamate/malate, palmitoyl carnitine/malate, or succinate/rotenone were significantly and proportionately diminished in *Sesn2*^−/−^ cells as compared with *Sesn2*^+/+^ controls (Fig. 7e). Similarly diminished uncoupler-stimulated rates were observed on each of the substrates (data not shown). To examine the potential impact of Sesn2 on mitochondrial respiration in the conditions of glucose deprivation we incubated cells in glucose-free medium for 6 hr and analyzed basal and maximal mitochondrial respiration as described above. We found that basal and maximal levels of mitochondrial respiration were significantly diminished in Sesn2-deficient MEF as compared to control cells (Fig. 7f,g). However, despite the compromised mitochondrial activity, the amounts of mitochondrial DNA were higher in Sesn2-deficient MEF incubated with control and glucose-free medium (Fig. 7h) as compared to Sesn2-proficient MEF, indicating that the effects of Sesn2-inactivation on mitochondrial activity cannot be simply explained by regulation of mitochondrial content by Sesn2. Altogether, we conclude that Sesn2 plays an important role in supporting mitochondrial function that can protect cell viability in conditions of nutrient deficiency.

## Discussion

Glucose is a significant substrate for ATP production and glycosylation for eukaryotic cells. Glucose deficiency, induced by normal processes of tissue growth during embryogenesis or by pathological conditions such as cancer, stroke, or infarction, stimulates the UPR^1^,^3^. The outcome of UPR activation depends on the severity and duration of ER stress: while the acute response potentially mitigates the consequences of ER stress, prolonged ER stress eventually stimulates cell death^4^,^5^. SESN2 has recently been reported as a protein activated by ER stress via ATF4, among the other potential UPR transcriptional factors^26^,^40^. NRF2 is also a critical regulator of SESN2 under various stress conditions^25^,^41^. Both of these transcriptional factors directly bind the *SESN2* promoter^25^,^26^ and are activated via the same PERK1-dependent branch of the UPR^3^,^7^. We have shown here that glucose starvation stimulates SESN2 expression via induction of ER stress and activation of both ATF4 and NRF2 followed by their direct binding to the *SESN2* promoter. These two transcription factors can interact and cooperate to activate the transcription of specific genes^42^,^43^. Interestingly, these effects were independent of the master regulator of SESN2, p53, which is critical for SESN2 activation by DNA damage^9^,^44^. Thus, we conclude that there are two major mechanisms of SESN2 activation: one via UPR-dependent activation ATF4 and NRF2, and another via p53 in response to DNA damage. In some stress conditions, such as that imposed by hydrogen peroxide treatment, both mechanisms may cooperate for maximal SESN2 induction^12^,^13^. In contrast to previous observations, SESN2 neither regulates expression of any UPR proteins, nor the activity of the AMPK-mTOR pathway during glucose withdrawal^28^,^29^,^45^. As reported previously, glucose deprivation does not induce autophagic flux^30^, and SESN2 plays no role in regulation of general autophagy under these conditions. As we demonstrated, SESN2 is a critical regulator of cell viability^9^,^12^,^24^. Accordingly, SESN2 inactivation strongly sensitizes cells to cell death induced by glucose starvation. Glucose starvation induces necrotic-like cell death associated with a drop of ATP levels, that prevents activation of the classical apoptotic program^46^. Despite the activation of caspases and production of ROS in response to glucose starvation, we did not see any significant role of caspases and ROS in cell death under our experimental conditions. Moreover, characterization of cell death by glucose starvation let us to rule out the impact of classical RIP1-dependent necroptosis, ferroptosis, and autophagy in cell demise induced by glucose starvation and rule out the potential contribution of Sesn2 in these processes. Thus, we presumed that Sesn2 regulates a non-canonical necroptotic cell death. Pursuing the potential mechanism of the regulation of cell death by Sesn2, we found that Necrostatin 7 suppresses cell death in *Sesn2*^−/−^ cells, equalizing it with the levels of cell death in *Sesn2*^+/+^ cells in response to glucose starvation. Necrostatin 7 is a recently described inhibitor of RIP1-independent necroptosis^38^. Thus, we concluded that Sesn2 is involved in protecting cells from necroptosis associated with glucose starvation. In agreement with this conclusion, the pro-survival role of Sesn2, we demonstrated recently that Sesn2 partially protects from necrotic cell death in the heart^47^. While this manuscript was under preparation, another group reported SESN2 as glucose-regulated gene that protects from apoptotic cell death in human hepatocarcinoma cells^45^. However, in contrast to their results, we did not see any role of SESN2 in the regulation of caspase-dependent apoptosis induced by glucose starvation, but demonstrated its role in necroptosis. Also, in contrast to their publication, we did not observe any impact of the AMPK-mTORC1 pathway or ROS on the regulation of cell viability by Sesn2 in response to glucose starvation. These differences might be explained by the involvement of SESN2 in different types of cell death in different cellular contexts. The potential mechanism of regulation of cell death by SESN2 in the conditions of energy stress might involve regulation of metabolism and ATP production. Mitochondria are critical source of ATP (required for apoptotic cell death) and insufficient ATP supply can re-direct cells to necrosis/necroptosis^48^,^49^. Accordingly, a drop in ATP levels occurred much faster in *Sesn2*^−/−^ cells as compared to *Sesn2*^+/+^ controls. The rate of ATP production on either endogenous stored substrates or alternative substrates present in DMEM (including glutamine, pyruvate, and other amino acids) may provide fuel necessary for cell survival under glucose starvation. We also observed that both the rate of extracellular acidification (proton production rate) and basal and maximal uncoupler-stimulated mitochondrial oxygen consumption are lower in the *Sesn2*^−/−^ fibroblasts under control conditions. Moreover, we observed that both basal and maximal levels of mitochondrial respiration were lower in glucose-starved *Sesn2*^−/−^ cells as compared to the control, thus contributing to diminished ATP production. The fact that rates of State 3 (phosphorylating) respiration on a variety of oxidizable substrates were all lower in the permeabilized Sesn2-deficient cells suggests diminished mitochondrial respiratory capacity in these cells independent of the substrate pathway. Together these data strongly suggest a role of Sesn2 in the control of overall cellular energy metabolism and maintenance of mitochondrial respiratory capacity. Further, the combination of intact and permeabilized cell respiratory data indicate that the mechanism of Sesn2 regulation does not involve selective inhibition of Complex I or II activity (Fig. 7e), nor does it specifically inhibit the enzymes and transporters directly involved in ATP synthesis, as uncoupler-stimulated respiration was also compromised (Fig. 7d,e). Nevertheless, we did not see a physical association of Sesn2 with mitochondria^12^,^20^, we and others recently described that SESN2 interacts with GATOR2^21^,^20^,^50^. Although the precise localization and function of GATOR2 remains unknown, SEACAT, the yeast analog of mammalian GATOR2, interacts with mitochondrial proteins and potentially regulates mitochondrial function^51^,^52^. In future work the role of GATOR2 in the regulation of mitochondrial activity and cell death by SESN2 will be explored. Another potential explanation for the role of SESN2 in regulation of mitochondrial function is the potential role of SESN2 in control of protein lysosomal degradation. It was demonstrated that SESN2 inactivation compromises lysosomal degradation of PDGFRβ and XIAP proteins^53^,^54^. Thus, the impact of SESN2 on the regulation of mitochondrial function and suppression of necroptosis might be mediated by control of degradation by a yet-to-be identified protein involved in the regulation of mitochondrial metabolism.

Necrosis/necroptosis contributes to the pathogenesis of many human diseases such as ischemic stroke, traumatic brain injury, neurodegenerative disorders and cancer^55^. Pharmacological modulation of SESN2 activity can be a powerful tool to prevent necrotic cell death under different pathological conditions and mitigate consequences of the diseases.

## Materials and Methods

### Cell culture, transfection, infection and treatment

Immortalized *Sesn2*^+/+^ and *Sesn2*^−/−^ MEF, and human lung adenocarcinoma H1299 and H460 cell lines were cultured in high-glucose DMEM containing 10% FBS and penicillin/streptomycin. H1299 cells were infected with sgCtrl.- or sgSESN2-bearing lentiviruses and selected with puromycin for two weeks to obtain stable Sesn2-deficient cell lines. H1299-tet-off-p53 cells were previously described^9^. All transfections were performed with Lipofectamine and Plus reagents (Life Technologies) and infections with lentiviral vectors were performed as previously described^14^. The treatments with glucose-free medium, 2-deoxyglucose, rotenone, Aicar, tunicamycin, N-acethyl-cysteine, Boc-D-FMK, rapamycin, 3-Methyladenine, chloroquine, Necrostatin 1, Ferrostatin 1, TUDCA, Necrostatin 7, were performed for 24 hr unless otherwise specified.

### Cell lysis and immunoblot analyses

Cells were lysed in RIPA-SDS buffer, after which the proteins were resolved by SDS-PAGE, transferred onto PVDF membranes, and probed with the relevant antibodies as previously described^9^. The following antibodies were used for the experiments: anti-actin (Sigma); anti-SESN2 (Proteintech), anti-NRF2, anti-CHOP and anti-p53 (Santa Cruz); and anti-phospho(T389)-p70S6K, anti-phospho(S235/236)-S6, anti-phospho(S65)-4E-BP1, anti-phospho IF2α(S51), anti-phospho(T172)-AMPKα, anti-phospho(S79)-acetyl-CoA carboxylase, anti-phospho(S9)-GSK3β, anti-phospho(T1462), anti-4E-BP1, anti-S6, anti-TSC2, anti-AMPKα, anti-ATF4, anti-Bip, anti-p62, anti-LC3, anti-PARP, anti-PARP(cleaved), anti-caspase3, and anti-caspase3(cleaved) (Cell Signaling Inc). Sesn1 antibodies were previously described^11^.

### Constructs

Reconstitution experiment was performed with pBabe-hygro Sesn2 (mouse) construct. The sequence for shSesn2 is 5′- GAAGACCCTACTTTCGGAT-3′ and for sgSesn2 is 5′-CTCGGAGTCCGCCACGATCA-3′. The shNRF2 and shATF4 were previously described^29^,^56^. The primers used for qPCR were as follows: SESN1: 5′-GCATGTTCCAACATTTCGTG-3′ and 5-TCCCACATCTGGATAAAGGC-3′; SESN2: 5′-GACCATGGCTACTCGCTGAT-3′ and 5′-GCTGCCTGGAACTTCTCATC-3′; and SESN3: 5′-ATGCTTTGGCAAGCTTTGTT-3′ and 5′-GCAAGATCACAAACGCAGAA-3′.

### Cell death analysis

Cells were treated, harvested and washed in PBS. Pellets were re-suspended in Annexin V binding buffer and incubated with anti-Annexin V FITC antibody and propidium iodide (PI) following the manufacturer’s instructions (BD Biosciences). Cells were acquired on a BD FACSCalibur instrument and data were analyzed with FCS Express 4 software (De Novo Software).

### ROS examination

To examine ROS levels, cells were incubated with DCF-DA for 30 minutes (Life Science) and analyzed by flow cytometry.

### ATP determination

ATP levels were examined with an ATP determination kit (Life Technologies A22066) according to manufacturer protocol. The cellular lysates were normalized to protein concentration and ATP concentration in the solution was measured per amount of lysate containing 1 μg of protein.

### Real Time PCR

RNA was isolated from Trizol-lysed cells and reverse-transcribed using Quanta Biosciences cDNA Synthesis kit. cDNA, primers, and SYBRGreen master mix (BioRad) were mixed and run on a Stratagene MX3000 instrument. Ct values were normalized using two housekeeping genes.

### Chromatin immunoprecipitation analysis (CHIP)

H1299 cells were cultured in regular medium or in glucose-free medium for the indicated time points, cross-linked with 1% paraformaldehyde, and harvested. Cells were then lysed and protein-chromatin extracts were obtained and digested using the Cell Signaling Technology ChIP kit (catalog # cs-9005) as per the manufacturer’s instructions. ChIP grade Abs for anti-histone H3 (cs-4620), anti-NRF2 (cs-12721), anti-ATF4 (cs-11815), or normal rabbit IgG (cs-2729) were incubated over night with the cell extracts. Magnetic beads were then added and protein-chromatin-Ab immune complexes were precipitated. After reversing the cross-links, DNA was purified and targets were amplified by real time PCR using the following sets of primers specific for human *SESN2* promoter: NRF2: 5′-CAATAGGGATTGAGGTTCCAC-3′ and 5′-ACAGGGAATTCACCTTATTTGT-3′^25^; ATF4 distal: 5′-GATCCAATTGGCTGACTTTG-3′ and 5′-ACTAACACATTTGCTTGTTCAC-3′; and ATF4 proximal: 5′-ATGACTCTTAGGGCTGTCA-3′ and 5′-GGAATTCTGGGAGTTGTAGTC-3′^26^. The amount of immunoprecipitated DNA in each sample is represented as signal relative to the total amount of input chromatin.

### Analysis of Mitochondrial Respiration and Extracellular Acidification

*Sesn2*^+/+^ and *Sesn2*^−/−^ MEF were plated in XF96 plates at 2 × 10^4^ cells/well for 24 hr growth or at 1.25 × 10^4^ cells/well for 48 hr growth. On the day of the assay, growth medium was exchanged for bicarbonate-free DMEM (Sigma #5030) supplemented with 10 mM glucose, 3 mM glutamine, 1 mM pyruvate, and 2 mM HEPES. Rates of mitochondrial and maximal respiration in intact cells were measured and defined as previously reported^39^. Proton production rates were calculated after determining the medium buffering capacity for the 2.28 μL measurement chamber according to Mookerjee *et al*.^57^. State 3 respiration rates in permeabilized cells were measured using recombinant perfringolysin O (commercially XF PMP)^58^. Concentrations and protocols are given in Divakaruni *et al*.^59^. Oxygen consumption and proton production rates were normalized to relative cell number by post-hoc nuclear staining with CyQuant (ThermoFisher). In general, the nuclei counts across each cell type did not vary more than +/− 5%. To analyze cell respiration in glucose-starved cells, the cells were incubated with glucose free medium for 6 hr, and examined by Seahorse analysis in the glucose-free assay medium containing 1 mM pyruvate and 2 mM glutamine.

### Quantification of mitochondrial:genomic DNA ratio

MEF were cultured in the indicated conditions, washed twice with ice-cold PBS, and then lysed in 500 μl of lysis buffer (10 mM Tris pH8.0, 100 mM NaCl, 10 mM EDTA, 0.5% SDS, 100 μg/ml proteinase K) overnight at 55 °C. Next, 50 μl of 3 M sodium acetate was added to the tubes and mixed. DNA was precipitated by adding 500 μl/tube of 100% isopropanol, followed by a 2 hr incubation at −20 °C. DNA was pelleted by centrifuging samples at maximum speed in a microfuge for 20 minutes and was then washed twice in 70% ethanol. DNA was dissolved in 50–100 μl of H_2_O and its concentration was established using a NanoDrop 1000 spectrophotometer (ThermoScientific).

For real time PCR, 15 ng of DNA per reaction were amplified using the following primers - for mitochondrial DNA: Cytb F: GGAACAACCCTAGTCGAATG, Cytb R: AGGGCCGCGATAATAAATG; for genomic DNA: B2m F: CCTTAAGTCAAGGTGGTTATGA, B2 m R: GACTTTGGTAGTTACTAGTTATCCT. Ct values obtained at the end of the PCR were used to determine the relative mtDNA:gDNA ratio in each sample. mtDNA:gDNA ratio from the WT, untreated control was set as 1 and ratios from the other samples were then normalized to it.

### Statistical analysis

Statistical analysis was performed using two-sample t-test. For experiments involving more than two factors, a one-way or two-way ANOVA model was fit followed by linear contrasts to compare groups of interest and multiple comparisons were adjusted using the Bonferroni method. Statistical significance was defined as p < 0.05. Results are presented as mean ± standard deviation (±S.D.) of at least three independent experiments.

## Additional Information

**How to cite this article**: Ding, B. *et al*. Sestrin2 is induced by glucose starvation via the unfolded protein response and protects cells from non-canonical necroptotic cell death. *Sci. Rep.* **6**, 22538; doi: 10.1038/srep22538 (2016).
